# Gallic Acid Induces S and G2 Phase Arrest and Apoptosis in Human Ovarian Cancer Cells In Vitro

**DOI:** 10.3390/app11093807

**Published:** 2021-04-23

**Authors:** Zhiping He, Xingquan Liu, Fenghua Wu, Shaozhen Wu, Gary O’Neal Rankin, Ivan Martinez, Yon Rojanasakul, Yi Charlie Chen

**Affiliations:** 1 The Key Laboratory for Quality Improvement of Agricultural Products of Zhejiang Province, College of Agriculture and Food Science, Zhejiang A & F University, Lin’ an, Hangzhou 311300, China; 2 College of Health, Science, Technology and Mathematics, Alderson Broaddus University, Philippi, WV 26416, USA; 3 Department of Biomedical Sciences, Joan C. Edwards School of Medicine, Marshall University, Huntington, WV 25755, USA; 4 Department of Microbiology, Immunology & Cell Biology and WVU Cancer Institute, West Virginia University, Morgantown, WV 26506, USA; 5 Department of Pharmaceutical Sciences, West Virginia University, Morgantown, WV 26506, USA

**Keywords:** ovarian cancer, apoptosis, gallic acid, cell cycle arrest

## Abstract

Ovarian cancer (OC) is among the top gynecologic cancers in the US with a death tally of 13,940 in the past year alone. Gallic acid (GA) is a natural compound with pharmacological benefits. In this research, the role of GA on cell proliferation, cell apoptosis, cell cycle-related protein expression was explored in OC cell lines OVCAR-3 and A2780/CP70. After 24,48 and 72 h of GA treatment, the IC50 values in OVCAR-3 cells were 22.14 ± 0.45, 20.36 ± 0.18, 15.13 ± 0.53 μM, respectively and in A2780/CP70 cells IC50 values were 33.53 ± 2.64, 27.18 ± 0.22, 22.81 ± 0.56, respectively. Hoechst 33,342 DNA staining and flow cytometry results showed 20 μM GA exposure could significantly accelerate apoptosis in both OC cell lines and the total apoptotic rate increased from 5.34%(control) to 21.42% in OVCAR-3 cells and from 8.01%(control) to 17.69% in A2780/CP70 cells. Western blot analysis revealed that GA stimulated programmed OC cell death via a p53-dependent intrinsic signaling. In addition, GA arrested cell cycle at the S or G2 phase via p53-p21-Cdc2-cyclin B pathway in the same cells. In conclusion, we provide some evidence of the efficacy of GA in ovarian cancer prevention and therapy.

## 1. Introduction

Ovarian cancer (OC) is among the top gynecologic cancers in the US with a death tally of 13,940 in the past year alone [1]. With each passing year, 239,000 new cases emerge worldwide, along with 152,000 fatalities [2]. In other words, ≥2/3 of the ovarian cancer patients will die from the disease. Unfortunately, this statistic has remained the same over the last 30 years. One possible reason may be the fact that, in only 17% of the cases, OCs are detected while it is localized in the ovaries. In a vast majority (>62%) of cases, the disease is diagnosed after the malignancy has spread to distant locations [3]. Despite advancements in OC therapy, including surgery and platinum-based chemotherapy, the 5- and 10-year survival periods of late stage cancer patients are only 32% and 15%, respectively [4]. OC is very sensitive to chemotherapy. However, after an initial good response to treatment, patients often relapse, requiring a multidrug approach, which introduces numerous adverse effects [5–7]. Therefore, it is crucial and urgent to develop new therapeutics for OC treatment and prevention, possibly from natural compounds, so as to manage OC.

Gallic acid (3,4,5-trihydroxybenzoic acid, GA) is the principal polyphenol in tea, grapes, berries and other fruits, and wine. Multiple reports have suggested GA to have antibacterial, antiviral, and anti-inflammatory properties. Its anti-tumor activity has been reported in breast [8], lung [9], oral [10], prostate [11] and cervical cancer cells [12].

In prior studies, we demonstrated that GA produced the largest suppression of human OC cells, among eight natural phenols used in traditional Chinese medicine (TCM) [13]. More specifically, we revealed that GA selectively inhibited two separate cell lines of OC, but not normal ovarian cells (IOSE 364 cells) [14]. Moreover, GA ceased VEGF secretion and reduced angiogenesis via the PTEN-AKT-HIF-1*α*-VEGF signaling network in a concentration-dependent manner. In animal experimentation, GA was shown to reduce tumor development by 50%, as evidenced by diminished vascularity, necrosis/fibrosis, neoplastic stromal retraction, and apoptosis [15]. Apoptosis is the main physiological phenomenon of natural compounds inhibiting the development of tumor cells. However, there is no report on GA-induced apoptosis of OC cells and its underlying mechanism. In this research, the suppressive role of GA on OC proliferation, apoptosis and cell cycle was explored. OVCAR-3s and A2780/ CP70s are two platinum-resistant ovarian cancer cell lines. OVCAR-3s has a wild-type p53 gene sequence while A2780/ CP70s has a point mutation in the p53 gene [16]. Particularly, this study focused on the GA-mediated apoptosis of OVCAR-3 cells.

## 2. Materials and Methods

### 2.1. Cell Culture and Reagents

The human OC cell lines OVCAR-3 and A2780/CP70 were kind gifts from Dr. Bing-Hua Jiang at Thomas Jefferson University, PA, USA. Cells were grown in RPMI 1640 medium (Sigma, St. Louis, MO, USA) with 10% fetal bovine serum (FBS) (Invitrogen, Grand Is-land, NY, USA), in a humid incubator with 5% CO_2_ at 37 °C.

Dead Cell Apoptosis Kits with Annexin V AlexaFluor^®^ 488 and propidium iodide (PI) was obtained from Thermo Fisher Scientific (Waltham, MA, USA). Caspase-Glo^®^ 3/7 Assay Systems, CellTiter 96^®^ AQueous One Solution Cell Proliferation Assay was obtained from Promega (Madison, WI, USA). Antibodies against Bax (2772S), cleaved caspase-3 (Asp175) (9654S), p21 Waf1/Cip1 (2947P), Cdc2 (9112P), p-Cdc2 (Tyr15) (9111P), and cyclin B1 (4138P), were obtained from Cell Signaling Technology, Inc. (Danvers, MA, USA). Primary antibodies against p53 (C-11) (sc-55476), Bad (C-7) (sc-8044), and GAPDH (0411) (sc-47724) were obtained from Santa Cruz Biotechnology Inc. (Santa Cruz, CA, USA). 100 mM GA was made in DMSO and maintained at −20 °C. Subsequent concentrations of GA were made in RPMI 1640 medium and DMSO (i.e., all GA concentrations contained the same concentration of DMSO, both compounds were bought from Sigma, St. Louis, MO, USA).

### 2.2. Assessment of Cell Viability

Both OC cell lines were plated in 96-well plates (1 × 10^4^ cells/well) with RPM-1640 + 10% FBS and maintained at 37 °C for 16 h. Next, the medium was replaced to include varying concentrations of GA, namely, 5, 10, 20, 40 μM, or DMSO as a control. The cells were further incubated for 24 h, 48 h, and 72 h. Subsequently, the cells were phosphate-buffered saline (PBS)-washed and exposed to 100 μL AQueous One Solution (MTS tetrazolium compound) for 1 h at 37 °C before absorbance reading at 490 nm, using a microplate reader.

### 2.3. Hoechst 33342 Staining

Both OC cell lines were plated in 96-well plates (1 × 10^4^ cells/well) with RPM-1640 + 10% FBS and maintained at 37 °C overnight. Upon treatment with GA (5, 10, 20, 40 μM) or DMSO for 48 h, the cells were stained using 10 μg/mL Hoechst 33342 (Sigma, St. Louis, MO, USA) in PBS for 15 min in the dark at 37 °C. After that, cells were assessed by fluorescence microscopy (Carl Zeiss, Heidelberg, Germany) in a blinded manner to avoid experimental bias. Apoptotic effect was evaluated through morphological changes.

### 2.4. Apoptosis Analysis by Flow Cytometry

Both OC cell lines were exposed to GA (5, 10, 20, 40 μM) or DMSO control for 48 h. Next, the cells were stained with Annexin V Alexa Fluor^®^ 488 and propidiumiodide (PI), following manufacturer’s guidelines. Lastly, the stained cells were then analyzed by FACSCalibur flow cytometry (BD Biosciences, San Jose, CA, USA) with fluorescence emission at 530 nm and excitation at 488 nm.

### 2.5. Detection of Caspase-3/7 Enzyme Activities

Cells (1 × 10^4^ OVCAR-3) were plated in 96-well plates and maintained O/N at 37 °C with 5% CO_2_ overnight. Next, the cells were exposed to GA (5, 10, 20, 40 μM) or DMSO control for 48 h. Caspase-Glo 3/7 Assay kit (Promega) were supplemented and incubated for 30 min. Luminescence was measured through the use of a microplate reader (BioTek, Winooski, VT, USA). Analyses were performed three times and results were conveyed as a proportion of controls.

### 2.6. Western Blot

Cells (1 × 10^6^ OVCAR-3) were plated in 60-mm dishes and cultured O/N prior to GA (control, 5, 10, 20 μM) exposure. Next, they were PBS-washed 1X, lysed in 100 μL mammalian protein extraction reagent including 1μL Halt Protease, 1 μL phosphatase inhibitor, and 2 μL ethylenediaminetetraacetic acid (EDTA) at room temperature. The isolated proteins were then separated on a 10% SDS-PAGE gel electrophoresis, and transferred to a nitrocellulose membrane using the Mini-Protean 3 System (Bio-Rad Laboratories, Hercules, CA, USA), followed by blocking with 5% nonfat milk in Tris-buffer with 0.1% Tween-20 for 1 h (room temperature, RT), and subsequent incubation with primary overnight (1:1000 dilution) and secondary antibodies (1 h), following standard protocols, TBST-wash, and exposure to Super Signal West Dura Extended Duration Substrate (Pierce) antigen-antibody for development of color and luminescence. Lastly, the protein levels were quantified using NIH ImageJ software 1.47 (NIH, Bethesda, MD, USA) and normalized to endogenous GAPDH concentrations.

### 2.7. Cell Cycle Evaluation Using Flow Cytometry

The test method of Cell cycle evaluation was according to our previous procedure [17]. In brief, after exposure with GA (control, 5, 10, 20 μM) for 48 h, the cells were trypsin-digested, centrifuged at 3000× *g* rpm for 10 min, PBS-washed, suspended in 70% ethanol, and stored at −20 °C. For cell cycle evaluation, the stored cell suspension was centrifuged at 1000× *g* rpm for 6 min before re-suspension in PBS, and centrifuged again to collect pellet, and finally exposed to 180 μg/mL RNase A at 37 °C for 15 min and 50 μg/mL propidium iodide stain for 15-min before flow cytometry (FACSCalibur system, BD Biosciences) evaluation. Data analysis was done with FCS software (De Novo Software, Los Angeles, CA, USA).

### 2.8. Statistical Analysis

All data are an average of more than three separate experiments ± standard deviation (SD). Data analysis was completed with one-way analysis of variance (ANOVA) with LSD’s test by SPSS software (IBM, Version 22.0, Armonk, NY, USA). Statistical significance was set at *p*-value of < 0.05.

## 3. Results

### 3.1. GA Inhibition of OC Cell Viability Is Dose Dependent

**Figure 1.**
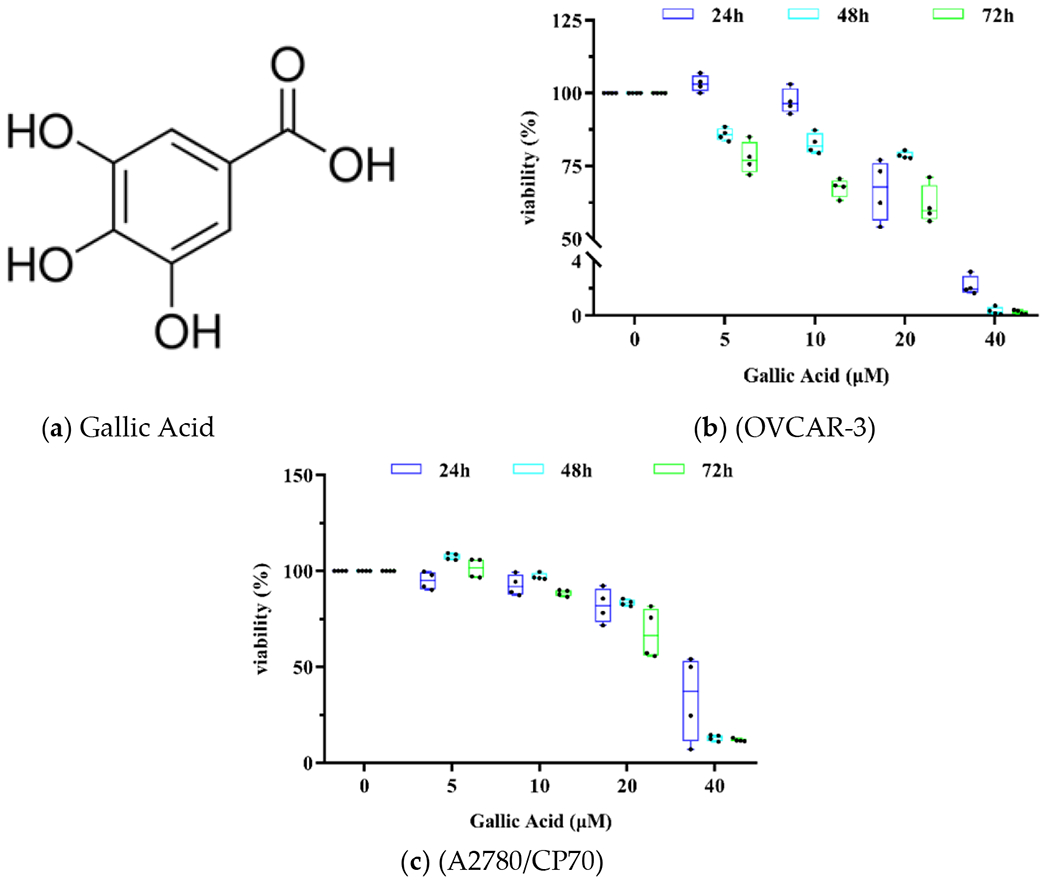
GA suppresses OC cell proliferation. (**a**) Structure of GA. (**b**) GA inhibited OVCAR-3 cell viability. (**c**) GA inhibited the A2780/CP70 cell viability.

To explore the antiproliferative role of GA on human OC cell lines OVCAR 3 and A2780/CP70, a cell proliferation assay was carried out. Antiproliferative effects of GA (0, 5, 10, 20 and 40 μM) were evaluated at 24, 48, and 72 h post exposure (Figure 1). The cell viability of OVCAR 3 at 40 μM were 2.19 ± 0.70%, 0.33 ± 0.28%, 0.27 ± 0.16%, respectively. The cell viability of A2780/CP70 at 40 μM were 33.94 ± 22.16, 13.09 ± 1.62, 11.90 ± 0.80, respectively. These results indicated that GA strongly suppressed both OC cell line viabilities and it was dose dependent. Upon 24, 48, and 72 h of GA exposure in OVCAR 3 cells, IC50 values were 22.14 ± 0.45, 20.36 ± 0.18, 15.13 ± 0.53 μM, respectively. The A2780/CP70 cells exhibited higher viability than OVCAR 3 cells with IC50 values at 33.53 ± 10.54, 27.18 ± 0.88, 22.81 ± 2.23 μM, respectively.

### 3.2. GA Promotes Cell Apoptosis in Both OC Cell Lines

**Figure 2.**
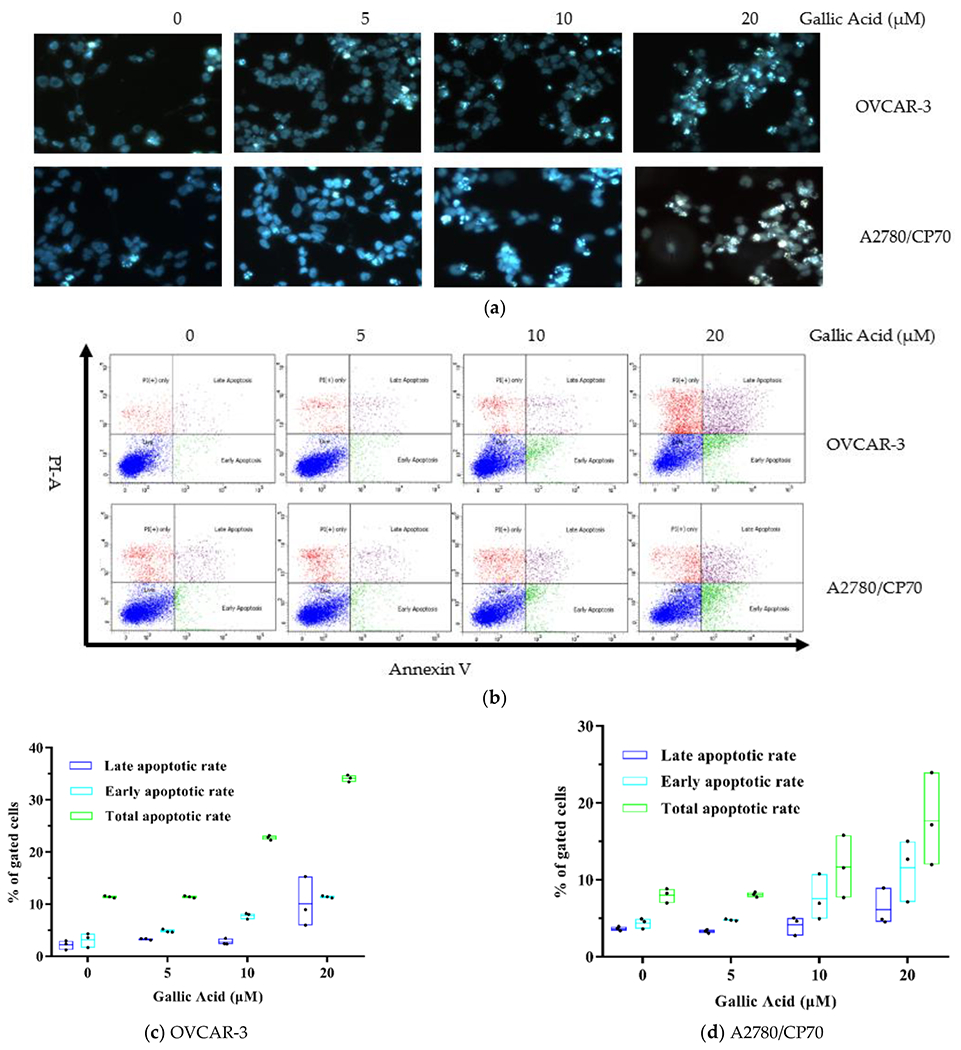
GA promotes OC cell apoptosis. (**a**) Cell viability evaluation of both OC cell lines after GA exposure for 48 h, using Hoechst 33342 staining and fluorescent microscopy (the apoptotic cells and total cells in Figure 2a were 4/35, 14/103, 21/69, 31/60 of OVCAR-3 cells and 6/58, 11/75, 19/65, 27/51 of A2780/CP70, respectively). (**b**) GA induced cell apoptosis in both OC cell lines, as evidenced by flow cytometry. (**c**) Effect of GA on late and early apoptotic rates and the total apoptotic rate in OVCAR-3 cells. (**d**) Outcome of GA on late and early apoptotic rates and the total apoptotic rate in the A2780/CP70 cell line.

To explore GA-mediated inhibition of OC cell viability via cell apoptosis, we exposed both OC cells lines to GA treatment (0, 5, 10, 20 μM) for 48 h and assessed nuclear morphology alterations using Hoechst 33342 DNA staining. We demonstrated marked chromatin condensation and nucleus shrinkage with GA exposure in both OC cell lines (Figure 2a). To further confirm these results, we performed flow cytometry assays via Annexin V/PI staining. Based on our results, GA exposure markedly elevated apoptotic cell population in both OC cell lines and it was dose dependent (Figure 2b). The total apoptotic rate rose to 21.42% with 20 μM GA in OVCAR-3 cells vs. 5.34% in control cells (Figure 2c) (*p* < 0.01). Moreover, relative to controls, the early and late apoptotic rates of the OVCAR-3 cell line, with 20 μM GA exposure, were also significantly high at 11.36% and 10.06% (*p* < 0.01) respectively. As with the viability results, the suppressive role of GA on A2780/CP70 cells was lower than on OVCAR-3 cells. Similarly, GA exposure also induced less apoptosis on A2780/CP70 cells than OVCAR-3 cells. The total apoptotic rate rose to 17.69% with 20 μM GA exposure in A2780/CP70 cells vs. 8.01% in control cells (Figure 2d) (*p* < 0.01). From Figure 2b, the fraction of dead but non-apoptotic (PI+ only) cells also increase in a dose-dependent manner for OVCAR-3 cells. This indicates some non-apoptotic effects induced by GA on OVCAR-3 cells. It is reported that Rhein derivative 4a RHA also causes non-apoptotic effects on ovarian cancer cells by endoplasmic reticulum stress [18]. The non-apoptotic mechanism induced by GA on OVCAR-3 cells is still unclear, and further research is needed.

### 3.3. GA Activates Caspase-3 in OVCAR-3s via the p53-Dependent Intrinsic Pathway

**Figure 3.**
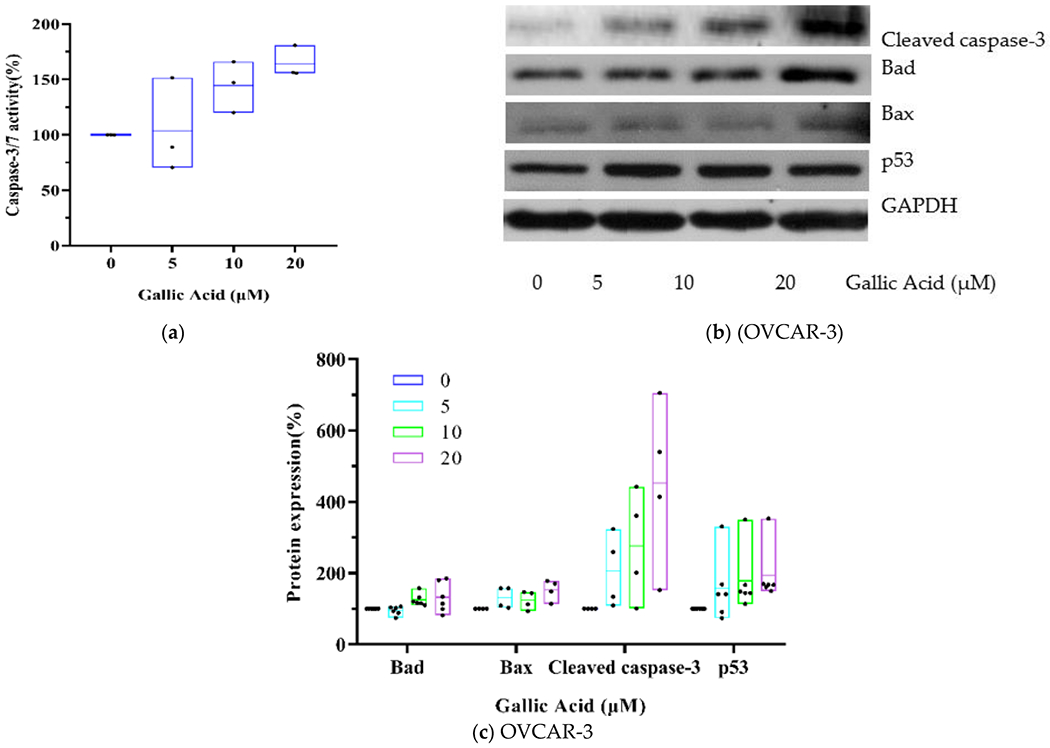
GA induces OVCAR-3 apoptosis by activating p53-dependent signaling. (**a**) Evaluation of Caspase 3/7 activity after 48 h exposure with GA. (**b**) Western blot image. (**c**) Protein expression quantization of Bad, Bax, cleaved caspase-3 and p53.

Caspases belong to the cysteine proteases family and are intricately involved in the apoptotic process. In fact, activating the “executioner” caspase-3 enzyme initiates the breakdown of major structural proteins within the cell [19]. Based on our results, GA exposure markedly increased Caspase-3/7 activity (Figure 3a). Moreover, pro-apoptotic proteins, including cleaved caspase-3 (4.53-fold), Bad (1.33-fold) and Bax (1.53-fold) were also markedly elevated, as shown in Figure 3b,c. The tumor sup-pressor p53 has been identified as an important mediator of apoptosis. In this research, compared to control, 20 μM GA exposure up-regulated p53 protein (1.95-fold) in OVCAR-3 cells (*p* < 0.05).

### 3.4. GA Causes S and G2 Phase Arrest in Both OC Cell Lines

To elucidate the underlying mechanism promoting GA-mediated OC cell viability suppression and apoptosis stimulation, we evaluated the consequence of GA exposure on the cell cycle of both OC cell lines. Both OC cell lines were exposed to t GA treatment (5, 10, 20 μM) or DMSO control for 48 h and the cell cycle profile was assessed with flow cytometry.

**Figure 4.**
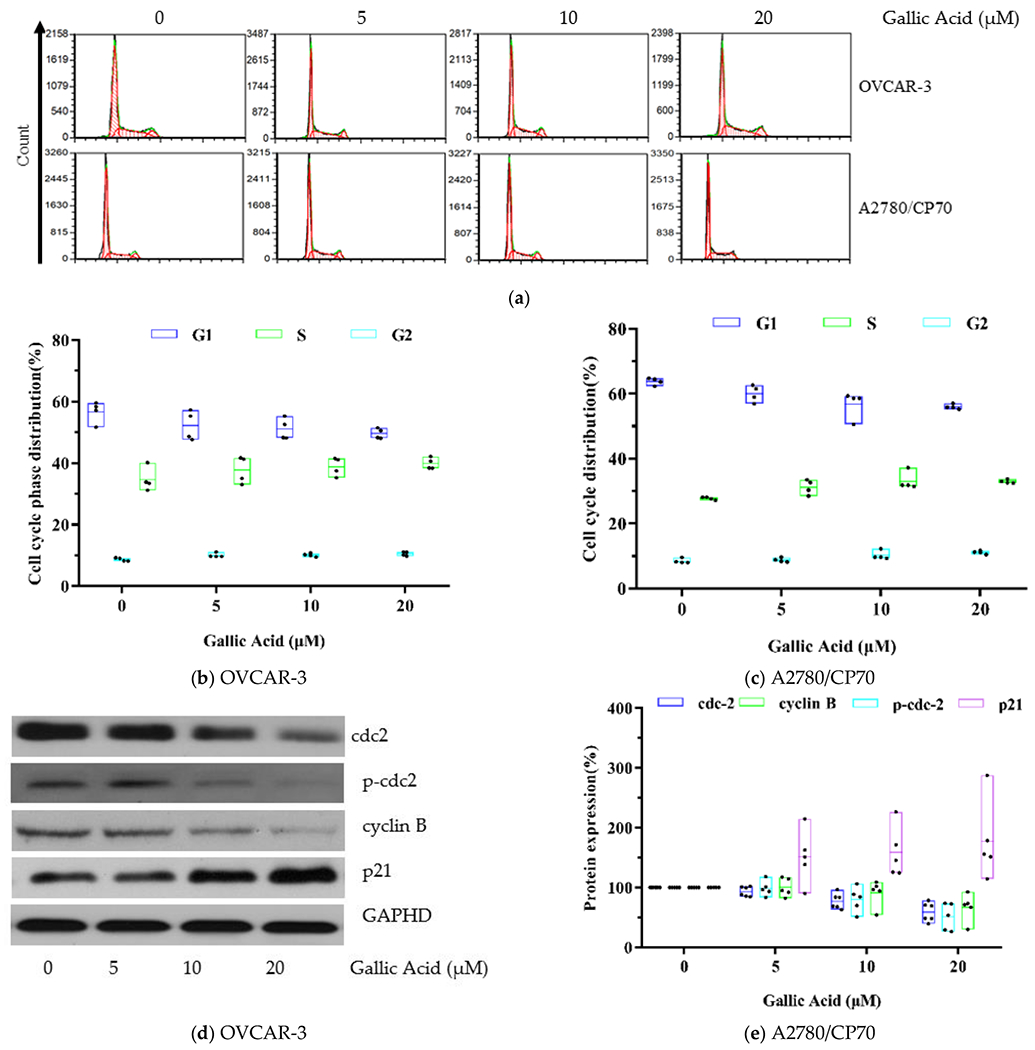
GA promotes phase arrest in OC cell lines. (**a**) Flow cytometry image. (**b**) GA stimulated larger population of cells at the S and G2 phase in OVCAR-3 cells. (**c**) GA stimulated larger population of cells at the S and G2 phase in A2780/CP70 cells. (**d**) Western blot image for OVCAR-3. (**e**) Protein expression quantization of cell cycle-related proteins for OVCAR-3.

The control, untreated OVCAR-3 cells showed 56.69% G1 phase, 34.65% S phase and 8.67% G2 phase. With GA treatment at 20 μM, the treated cells showed 49.62% G1 phase, 39.89% S phase and 10.49% G2 phase (Figure 4b). Based on these data, there was a small elevation in the % of OVCAR-3 cells in the S or G2 phases (*p* < 0.05), along with a simultaneous reduction in the % of OVCAR-3 cells in the G1 phase (*p* < 0.01). The cell cycle results of control, untreated A2780/CP70 cells showed 63.79% G1 phase, 27.76% S phase and 8.46% G2 phase. With GA treatment at 20 μM, the treated cells showed 55.95% G1 phase, 32.93% S phase and 11.13% G2 phase. Therefore, GA exposure also produced a small elevation in the % of /CP70s cells in the S or G2 phases (*p* < 0.01), along with a simultaneous decrease in the % of A2780/CP70s cells in the G1 phase (Figure 4c) (*p* < 0.01). Collectively, these data indicate that GA exposure can halt both OC cell line cell cycle at the S or G2 phase.

### 3.5. GA Modulates Cell Cycle-Related Proteins Levels in OVCAR-3s

To identify the underlying mechanism of GA-mediated cell cycle arrest, CDC2, p-Cdc2 (Tyr15), cyclin B, and p21 protein levels were evaluated using Western blot analysis. Based on our results, GA exposure significantly decreased the levels of cell cycle-related proteins, namely Cdc2 (0.59-fold), p-Cdc2 (Tyr15) (0.51-fold) and cyclin B (0.67-fold), shown in Figure 4e. p21 is a cyclin dependent kinase inhibitory protein which regulates cell cycle progression. In this research, compared to control, an up-regulation of p21 pro4.

## 4. Discussion

GA is a natural polyphenol with antibacterial, antiviral, and antitumor properties. Its tumor suppressive properties has, thus far, been demonstrated in prostate cancer [11], breast cancer [20], bladder cancer [21], cervical cancer and lung cancer [9].

In prior experiments, we demonstrated that GA produced the largest suppression of human OC cells, among eight natural phenols used in TCM [13,14]. In this paper, the IC50 values of GA exposure in human OC cell lines OVCAR 3 and A2780/CP70 with 24, 48, 72 h exposure were further evaluated. As in our previous experiments, the suppressive effects of GA on OVCAR-3 cells were stronger, relative to A2780/CP70 cells. The specific mechanism is still unclear. In addition, a decrease of IC50 values of GA in both cell lines was observed when the treatment time increased from 24 h to 72 h. Our findings are in accordance to previously published reports [22,23].

Many polyphenols inhibit the viability of cancer cells due to their induction of apoptosis [24]. Recent research showed that polyphenols induce apoptosis by suppress or activate mitochondrial death system and affect mitochondrial function and structure [25]. In this study, GA showed significant activity of apoptosis induction at 10 μM and 20 μM in OVCAR 3 and A2780/CP70 cells, respectively. In all, these data suggest that GA suppresses OC cell growth by promoting apoptosis. This form of apoptotic stimulation has been demonstrated in other cancerous cells. GA showed significant apoptosis induction at 10 μM in lymphoblastic leukemia cell line C121 [26] and at 50 μM in human breast cancer cell MDA-MB-231 [27].

Stimulating apoptosis in cells is a key mechanism by which anticancer therapy works. In addition, GA was also shown to have enhanced bioavailability in humans, relative to other polyphenols [28]. In fact, oral intake of dietary herbal supplement with 800 mg GA resulted in 8–10 μM GA in the serum of healthy volunteers [29]. According to results from these studies, GA demonstrated a strong potential as an anti-tumor agent.

The mitochondrial intrinsic pathway is the main pathway for polyphenols to induce cell apoptosis [30,31]. In this research, we demonstrated marked elevation in pro-apoptotic proteins; namely, cleaved caspase-3 (4.53-fold), Bad (1.33-fold) and Bax (1.53-fold). This result indicated that GA activated caspase-3 mediated intrinsic apoptotic pathways in OVCAR-3 cells via the upregulation of proapoptotic proteins (Bax and Bad). Tumor suppressor p53 protein modulates cell apoptosis [32]. Our previous research found by kaempferol, a natural flavonoid, induced intrinsic apoptosis via activation of p53 in OVCAR-3 cells [33]. To examine the effect of p53 on GA-mediated apoptosis, p53 protein levels was assessed via Western blotting. We demonstrated that the basal expression of p53 protein was markedly elevated (*p* < 0.05) post GA exposure in OVCAR-3 cells. This result suggests that the GA-mediated apoptosis occurs via the regulation of p53 protein. The p53 point mutation R248Q, found in OVCAR3 cells, affects DNA binding and has been shown to reduce the ability to induce apoptosis [34]. Therefore, the role of p53 gene in the GA-mediated apoptosis of OVCAR-3 cells needs to be further confirmed, such as the use of chip technology to determine the binding sites of p53 protein on the promoter of apoptosis genes. This is the first paper to document the mechanism of promoting apoptosis with GA in OC cells.

Cell cycle arrest is an important factor affecting cell growth. Our previous research found that many natural compounds such as saponins [17], proanthocyanidins [35] and theaflavin-3,3-digallate [36], inhibit the viability of cancer cells via promoting cell cycle arrest. In this research, cell cycle analysis revealed that GA stimulated an increase in the population of S and G2 phase cells in two examined OC cell lines. It is reported that the Cdc2/cyclin B pathway is related to G2 cell cycle arrest [37]. In this research, Cdc2, p-Cdc2 and cyclin B were decreased. This regulation indicated that the GA-mediated phase arrest in OVCAR 3 cells is related to the Cdc2/cyclin B pathway. Artificially increasing p21 stability can prevent the activation of cyclin B–Cdc2 and promotion of G2/M arrest [38]. Further, p21 protein increased in GA-treated OVCAR 3 cells. Additionally, p53 protein was also up-regulated in GA exposed OVCAR 3 cells. This indicates that the activation of p21 causing G2/M phase arrest in GA-treated OVCAR 3 cells is p53-dependent. Collectively, these data indicate that GA halts cell cycle at the S or G2 phase via the p53-p21-Cdc2-cyclin B pathway.

## 5. Conclusions

In summary, this study demonstrated that GA suppressed the cell growth of two separate OC cell lines through the induction of apoptosis. Moreover, we revealed that GA activates caspase-3 mediated intrinsic apoptotic pathways in OVCAR-3 cells via up-regulation of proapoptotic proteins (Bax and Bad) by p53 protein. GA also arrests the cell cycle at the S or G2 phase via the p53-p21-Cdc2-cyclin B axis. Taken together, GA can potentially be employed towards the treatment of OC. Considering that GA has good bioavailability, the function of GA needs further verification in vivo.
